# Recognizing Drug-Induced Methemoglobinemia in the Emergency Setting: A Case of Topical Dapsone Toxicity

**DOI:** 10.7759/cureus.92342

**Published:** 2025-09-15

**Authors:** Noor Ali, Mahmoud Abughazal, Razan Honeini, Rawan Honeini

**Affiliations:** 1 Emergency Medicine, United Lincolnshire Hospitals NHS Trust, Boston, GBR; 2 Emergency Medicine, United Lincolnshire Hospitals NHS Trust, Grantham, GBR

**Keywords:** acquired methemoglobinemia, dapsone-induced toxicity, emergency hypoxia, methemoglobin levels, oxidative drug reaction, oxygen desaturation, topical dapsone, unexplained cyanosis

## Abstract

Methemoglobinemia is an uncommon but important cause of hypoxia, most often triggered by exposure to certain medications or chemicals. Although rarely encountered in clinical practice, it can be life-threatening if not promptly recognized and treated. The condition occurs when methemoglobin levels in the blood exceed the normal physiological threshold of 1-2%. Even mild elevations can produce symptoms such as persistent headaches, dizziness, lethargy, and central cyanosis. As methemoglobin concentrations increase, the risk of severe hypoxic injury rises, with critically high levels capable of causing coma, arrhythmias, or death. We report the case of a 21-year-old previously healthy female who developed methemoglobinemia secondary to dapsone therapy. She presented with a two-week history of worsening headaches and palpitations, accompanied by several days of progressive shortness of breath. The absence of underlying cardiopulmonary or systemic disease initially complicated the diagnostic process. This case highlights the essential role of comprehensive history-taking in the evaluation of hypoxic patients, particularly younger individuals without apparent risk factors. A detailed review of the patient’s medication history ultimately identified dapsone use as the precipitating factor. This report serves as a reminder for clinicians to maintain a broad differential diagnosis when assessing unexplained hypoxia and to consider rare but reversible causes, such as methemoglobinemia, in the appropriate clinical context.

## Introduction

Methemoglobinemia is a rare hematological disorder characterized by elevated levels of methemoglobin, an oxidized form of hemoglobin in which the iron moiety exists in the ferric (Fe³⁺) state rather than the physiologically active ferrous (Fe²⁺) form. Under normal conditions, methemoglobin accounts for only 1-2% of total hemoglobin in red blood cells [1]. At higher concentrations, methemoglobin is unable to bind and deliver oxygen, resulting in functional anemia and tissue hypoxia. Clinically, cyanosis becomes apparent when methemoglobin levels reach approximately 15%, due to the pigment’s dark bluish-brown color [1,2]. As levels rise, patients may experience fatigue, headache, dyspnea, tachycardia, and, in severe cases, altered mental status and cardiovascular collapse. Concentrations exceeding 70% are rare but typically fatal without prompt intervention [3].

Methemoglobinemia may be congenital, most commonly resulting from deficiencies in enzymes such as cytochrome b5 reductase, but it is more frequently acquired. Acquired forms usually result from exposure to oxidizing agents, including certain drugs and chemicals [1,4]. Dapsone, a sulfone antibiotic with anti-inflammatory and antimicrobial properties, is used in both oral and topical formulations to treat dermatological conditions, notably acne vulgaris [2]. However, dapsone is a recognized oxidizing agent and has been implicated in the development of methemoglobinemia, with adverse effects reported following both oral and topical administration [3]. The pathophysiology involves the oxidation of hemoglobin induced by dapsone metabolites, leading to elevated methemoglobin levels and subsequent tissue hypoxia. Clinical recognition of this rare complication is crucial, particularly in patients presenting with unexplained cyanosis or hypoxemia unresponsive to oxygen therapy [5,6].

This case report describes a 21-year-old female who developed symptomatic methemoglobinemia following dapsone use. The case highlights diagnostic challenges, therapeutic considerations, and clinical implications, emphasizing the importance of including drug-induced methemoglobinemia in the differential diagnosis of unexplained hypoxia, especially in young, otherwise healthy individuals.

## Case presentation

A 21-year-old female presented to the emergency department with a two-week history of progressively worsening headaches, intermittent dizziness, and palpitations. Over the preceding three days, she had also developed exertional dyspnea, followed by resting dyspnea. Her medical history was notable for obstructive sleep apnea and acne vulgaris, for which she had been using spironolactone and topical dapsone 5% gel once daily for the past six months. She took no hormonal contraception and denied recent infection, travel, or exposure to toxins. She was not known to have G6PD deficiency.

On arrival, she was alert but in mild respiratory distress. The National Early Warning Score 2 was 6. Vital signs were heart rate 134 beats/min, respiratory rate 19 breaths/min, temperature 37.4°C, blood pressure 140/92 mmHg, and oxygen saturation 87% on room air. Chest auscultation was clear bilaterally, and no peripheral edema was observed. High-concentration oxygen therapy was initiated immediately. An arterial blood gas performed while the patient was receiving high-flow oxygen (Table 1) revealed type 1 respiratory failure, with a methemoglobin level elevated to 11.1%.

**Table 1:** Initial arterial blood gas analysis of the patient in the accident and emergency department

Parameter	Measured value	Normal range
pH	7.421	7.350-7.450
Carbon dioxide partial pressure (pCO₂)	4.96 kPa	4.30-6.40 kPa
Oxygen partial pressure (pO₂)	25.9 kPa	11.0-14.4 kPa
Hematocrit	31.30%	34.7-49.2%
Sodium	141 mmol/L	133-146 mmol/L
Potassium	3.9 mmol/L	3.5-5.3 mmol/L
Chloride	108 mmol/L	95-108 mmol/L
Ionized calcium	1.19 mmol/L	1.15-1.33 mmol/L
Glucose	5.9 mmol/L	3.0-6.0 mmol/L
Lactate	0.7 mmol/L	1.0-1.8 mmol/L
Total hemoglobin	102 g/L	117-170 g/L
Saturated oxygen (SpO₂)	98.50%	94.0-98.0%
Oxyhemoglobin	87.60%	94.0-98.0%
Carboxyhemoglobin	0%	0.0-3.0%
Methemoglobin	11.10%	0.0-1.5%
Deoxyhemoglobin	1.30%	0.0-3.0%
Actual base excess	-0.1 mmol/L	-2.0-2.0 mmol/L
Actual bicarbonate	24.2 mmol/L	22.0-29.0 mmol/L
Temperature	37°C	-
Fraction of inspired oxygen (FiO₂)	21%	-

Baseline laboratory studies revealed a white cell count of 12.2 × 10⁹ L⁻¹ (neutrophils 9.83 × 10⁹ L⁻¹) and a D-dimer level of <150 ng/mL. Blood cultures remained negative after five days. Electrocardiography demonstrated sinus tachycardia. The Wells score was 4.5, prompting discussion between the emergency medicine and medical teams and the arrangement of an urgent CT pulmonary angiogram (CTPA).

**Figure 1:**
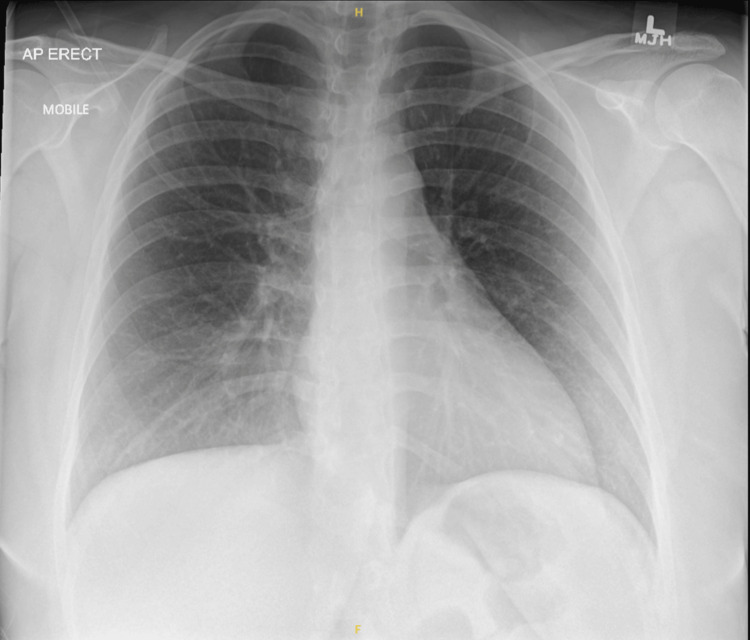
Normal chest X-ray of the patient

**Figure 2:**
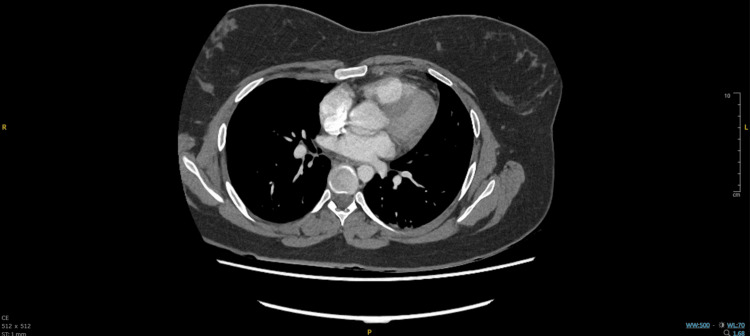
Normal contrast-enhanced CT of the pulmonary arteries (mediastinal window)

**Figure 3:**
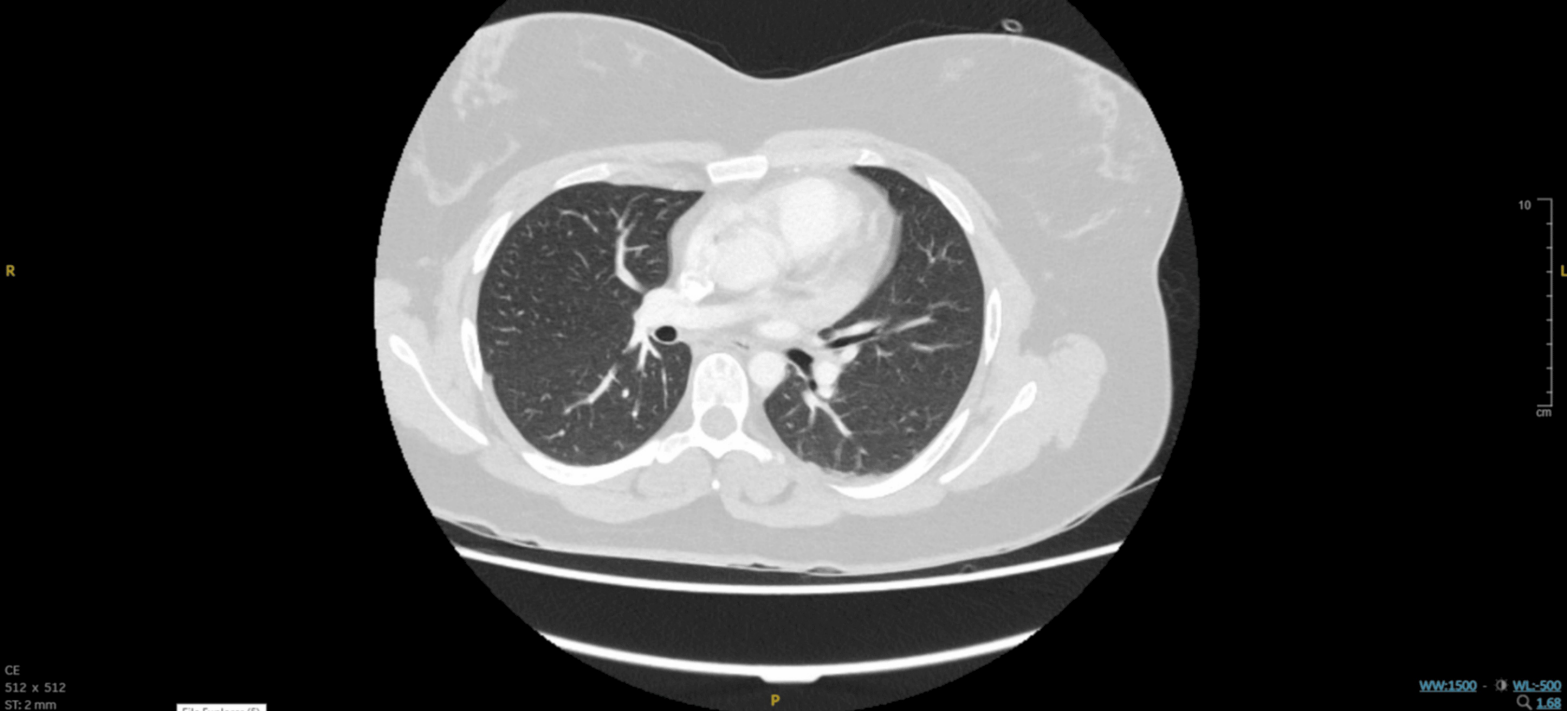
Normal contrast-enhanced CT of the pulmonary arteries (lung window)

A portable erect AP chest radiograph (Figure 1) showed clear lung fields with no consolidation, collapse, effusion, or pneumothorax; cardiac size and mediastinal contours were difficult to assess due to projection. CTPA images (Figure 2, Figure 3) revealed no filling defects within the central or segmental pulmonary arteries to suggest pulmonary embolism (PE) and no pleural or parenchymal pathology. Small-volume mediastinal lymph nodes were noted, while the visualized upper abdominal viscera appeared unremarkable.

The patient was admitted to the acute medical unit for close monitoring. Dapsone was discontinued, and she was managed with high-flow oxygen delivered via alternating Venturi and non-rebreather masks at up to 15 L/min. Serial venous blood gases demonstrated a steady decline in methemoglobin levels without rebound, with the final VBG in the medical ward showing a methemoglobin level of 1.9% (Table 2).

**Table 2:** Latest venous blood gas of the patient in the ward prior to discharge

Parameter	Measured value	Normal range
pH	7.379	7.350-7.450
Carbon dioxide partial pressure (pCO₂)	5.97 kPa	4.30-6.40 kPa
Oxygen partial pressure (pO₂)	5.35 kPa	11.0-14.4 kPa
Hematocrit	32.60%	34.7-49.2%
Sodium	142 mmol/L	133-146 mmol/L
Potassium	4 mmol/L	3.5-5.3 mmol/L
Chloride	106 mmol/L	95-108 mmol/L
Ionized calcium	1.23 mmol/L	1.15-1.33 mmol/L
Glucose	5.7 mmol/L	3.0-6.0 mmol/L
Lactate	1.1 mmol/L	1.0-1.8 mmol/L
Total hemoglobin	106 g/L	117-170 g/L
Saturated oxygen (SpO₂)	70.80%	94.0-98.0%
Oxyhemoglobin	69.10%	94.0-98.0%
Carboxyhemoglobin	0.50%	0.0-3.0%
Methemoglobin	1.90%	0.0-1.5%
Deoxyhemoglobin	28.50%	0.0-3.0%
Actual base excess	0.9 mmol/L	-2.0-2.0 mmol/L
Actual bicarbonate	26.4 mmol/L	22.0-29.0 mmol/L
Temperature	37°C	-
Fraction of inspired oxygen (FiO₂)	21%	-

By day 4, her oxygen saturation had normalized on room air, and she was weaned off supplemental oxygen. She was discharged on day 6, hemodynamically stable and asymptomatic. Causality was assessed using the Naranjo scale, yielding a total score of +6, indicating a probable adverse drug reaction to topical dapsone (Table 3).

**Table 3:** Naranjo Adverse Drug Reaction Probability Scale for this case

Naranjo Scale question	Answer	Score
1. Are there previous conclusive reports on this reaction?	Yes	1
2. Did the adverse event appear after the suspected drug was given?	Yes	1
3. Did the adverse reaction improve when the drug was discontinued?	Yes	1
4. Did the adverse reaction reappear when the drug was re-administered?	Not applicable	0
5. Are there alternative causes that could have caused the reaction?	No	2
6. Did the reaction reappear with the placebo?	Not applicable	0
7. Was the drug detected in any body fluid in toxic concentrations?	Not applicable	0
8. Was the reaction more severe when the dose was increased or less severe when decreased?	Not applicable	0
9. Did the patient have a similar reaction to the same or similar drugs in any previous exposure?	Not applicable	0
10. Was the adverse event confirmed by objective evidence?	Yes (arterial blood gas)	1
Total score		6

With infectious, cardiopulmonary, and thromboembolic causes excluded, the episode was attributed to dapsone-induced methemoglobinemia. The patient was counseled to avoid dapsone-containing products in the future and advised to seek prompt medical attention if similar symptoms recurred.

## Discussion

This case demonstrates that dapsone, even when applied topically for acne, can cause significant methemoglobinemia, a condition in which hemoglobin is oxidized into methemoglobin and loses its ability to carry oxygen effectively. This can lead to tissue hypoxia and, if untreated, may be life-threatening. The patient exhibited common symptoms of methemoglobinemia, including headache, dizziness, palpitations, and shortness of breath [1]. These symptoms reflected reduced oxygen delivery caused by elevated methemoglobin levels, confirmed by arterial blood gas analysis showing 11.1% methemoglobin. Due to low oxygen saturation and type 1 respiratory failure, oxygen therapy was initiated immediately, a critical step in managing this condition. Initially, a CTPA was performed to exclude PE, as the patient’s high Wells score, tachycardia, and hypoxia suggested PE despite normal D-dimer levels. When the scan revealed no PE and methemoglobinemia was confirmed, the diagnosis was revised accordingly.

Dapsone’s association with methemoglobinemia is well established [2,3]. The drug induces oxidative damage to red blood cells, raising methemoglobin levels, particularly in patients with certain risk factors or with higher doses. Although topical dapsone is generally considered safer, sufficient systemic absorption occurred in this case to cause harm, emphasizing the need for careful use and monitoring [7].

Treatment generally involves supplemental oxygen. In severe cases, intravenous methylene blue can be administered to convert methemoglobin back to functional hemoglobin. However, this patient improved with oxygen therapy alone, likely because her methemoglobin level was moderate [4,5]. Serial blood gas monitoring guided treatment and informed the tapering of oxygen support. This case underscores the importance of obtaining a detailed medication history and considering drug-induced causes when evaluating unexplained hypoxia [2,5]. Even topical dapsone, commonly used for acne, carries risks that patients should be counseled about, and careful follow-up is necessary.

Methemoglobinemia remains a rare but serious adverse effect of oxidizing agents such as dapsone [5,6]. It occurs because the drug converts hemoglobin into a form incapable of oxygen transport [7]. In this patient, clinical symptoms and arterial blood gas analysis confirmed the diagnosis, and oxygen therapy improved tissue oxygenation despite elevated methemoglobin levels [8]. The CT scan was crucial to rule out PE given the patient’s presentation, but the final diagnosis explained why the hypoxia did not align with other clinical findings, illustrating how methemoglobinemia can sometimes be overlooked [7,9].

Dapsone in acne treatment and risks

Dapsone is widely used for acne, both topically and orally [5,6]. Studies show that topical formulations reduce acne-related inflammation with fewer adverse effects than oral forms [10]. However, systemic absorption can vary, and levels may become sufficient to cause methemoglobinemia [7,11]. This case highlights that topical use is not entirely risk-free.

Managing methemoglobinemia

Management focuses on oxygen therapy to improve tissue oxygen delivery. When necessary, methylene blue accelerates the reduction of methemoglobin back to hemoglobin [12,13]. Although methylene blue is typically indicated for significant methemoglobinemia, it was not administered in this case. The patient responded well to high-flow oxygen alone, and her methemoglobin levels gradually declined with supportive care. The inpatient team opted against methylene blue, with no rationale documented in the clinical notes. The patient’s moderate methemoglobin level (11.1%) and stable cardiovascular status likely informed this conservative approach. This case demonstrates that some patients may improve without antidotal therapy, although methylene blue remains first-line for more severe or symptomatic cases.

This case emphasizes the importance of recognizing dapsone-induced methemoglobinemia, even from topical use. Careful medication review and clinical suspicion are essential when patients present with unexplained hypoxia [14].

## Conclusions

Dapsone, even when applied topically, can cause methemoglobinemia, resulting in significant hypoxia and related symptoms. Healthcare providers should remain aware of this rare but serious side effect in patients presenting with unexplained hypoxia, particularly those using dapsone. Early recognition and appropriate management are essential to prevent potentially life-threatening complications. Additionally, this case underscores the importance of educating patients about medication risks and ensuring regular monitoring during dapsone therapy.
